# A Novel Approach for Hybrid Image Segmentation GCPSO: FCM Techniques for MRI Brain Tumour Identification and Classification

**DOI:** 10.1155/2022/7453935

**Published:** 2022-12-23

**Authors:** P. Kavitha, Prabhu Jayagopal, M. Sandeep Kumar, Vetri Selvi Mahamuni

**Affiliations:** ^1^Department of Artificial Intelligence and Data Science, Panimalar Engineering College, Chennai, Tamil Nadu, India; ^2^School of Information Technology and Engineering, Vellore Institute of Technology, Vellore, Tamil Nadu 632014, India; ^3^Department of Management, College of Business and Economics, Mettu University, Metu, Ethiopia

## Abstract

In recent times, the early detection of brain tumour analysis and classification has become a very vital part of the medical field. The MRI scan image is the most significant tool to study brain tissue for proper diagnosis and efficient treatment planning to detect the early stages. In this research study, the two contributions were executed in the preprocessing mode. (a) Using wavelet transform to apply decomposed sub-bands of a low-frequency signal to control and adapt the spatial and intensity parameters in a bilateral filter and (b) to detect texture regions and block boundary to control and adapt the spatial and intensity parameters in a bilateral filter When compared to other image resolution methods, the adaptive bilateral method restores the original image quality and has a higher accuracy rate. Using the hybrid segmentation method of GCPSO (Guaranteed Convergence Particle Swarm Optimization) -FCM (Fuzzy *C*-Mean) techniques, the results were compared with various segmentation. The proposed segmentation gives a better accuracy rate of 95.32%.

## 1. Introduction

Early detection and treatment of these illnesses is extremely helpful to find out the abnormal cells in that image. The aim of the research process, MRI scan images were used to figure out that a person had a brain tumour. The MR images can be used to measure the size of the tumour cells. There are a number of ways to use segmentation to find brain tumour cells in an MRI scan image. On dynamic contrast-enhanced (DCE) MRI of the breast [1], a semiautomated and interactive technique based on the spatial Fuzzy C-Means (sFCM) algorithm is suggested [2]. The suggested method is used to obtain a segmented tumour region that is visible to the doctor and provides them with more information about the tumour for their diagnosis. The suggested method makes use of image filtering, threshold-based segmentation, morphological operations, and pixel subtraction. The suggested method is predicated on getting distinct images of the skull, brain, and tumour. When compared, the suggested strategy produced a better outcome than the alternative strategy.

The adaptive threshold-based technique, an alternative to a fixed threshold, can lessen these dependencies by considering the tumour to [3] background ratio (TBR) and tumour size. Because the parameters for the adaptive methods need to be calibrated for each PET camera system, it is difficult to employ the same methodology on different PET systems to get comparable results (e.g., scanner geometry, image acquisition protocol, and reconstruction methodology). Reports indicate that the performance of adaptive techniques underperforms for smaller volumes with lower TBR and SNR. Based on an improved direct region identification method, to [4] address MRgFUS post-treatment segmentation challenges for fibroid segmentation in MR images. In the recommended incremental process, an adaptive region-expanding procedure uses the split-and-merge method outcomes as various seed-region selections. The recommended technique isolates many fibroids with different pixel intensities, even within the same MR image. Utilizing area- and distance-based measures, the strategy was evaluated and compared to other studies of a similar kind in the literature.

Otsu thresholding, Discrete Wavelet Transform (DWT), Principal Component Analysis (PCA), Support Vector Machines (SVMs), Least Squared-Support Vector Machines (LS-SVMs), Proximal Support Vector Machines (PSVMs), and Twin Support Vector Machines (TWSVMs) are employed in the segmentation phase, feature extraction phase, feature reduction phase, and classification phase, respectively. The topic of creating active contour models and segmentation algorithms for automatically spotting brain cancers in multimodal volumetric MRI data. Using active contour and region-growing segmentation, we improved the detection of numerous brain tumours.

Comparing the suggested quantum-inspired classifier to traditional machine learning classifiers, it is a novel and superior methodology for evaluating clonogenic experiments [5]. It is shown to be an important feature in detecting difficult cell colonies among the extracted and analyzed image features. The 11 layers of the SegCNN model were developed using the Adam optimization solver and a predetermined batch size. The proposed model delivers a 98% dice similarity coefficient (DSC), which is noticeably superior to the most recent research reports. In the present research work, a diagnosis of brain tumour cell detection using several methods has been implemented for image denoising and image segmenting. For this research, the past ten years of research work were reviewed and discussed for comparison purposes. The proposed techniques are implemented and get more accurate results when compared with previous work. This work provides help to diagnose diseases in a very small period of time and with better accuracy and value, so this study will contribute more effectively in the ground of image processing. The research methodology of this work is as follows:

(1) The image database is generated by the collection of cancerous images from a private cancer hospital. 130 images are collected from private centers.
(2) To be developed a noise reduction algorithm using an Adaptive Bilateral Multiresolution technique that is more efficient in improving image resolution.
  1. A Adaptive Bilateral Multiresolution method is combined with a wavelet transform threshold.
  2. To eliminate compression artifacts using spatial adaptive methods and to avoid over-smoothing of original image texture regions while effectively removing ringing and blocking artifacts.
(3) Using a hybrid segmentation technique to segment a filtered image using GCPSO (Guaranteed Convergence Particle Swarm Optimization) and FCM (Fuzzy *C*-Mean) techniques was compared with various segmentations. The proposed segmentation gives a better accuracy rate of 95%.

**Figure 1:**
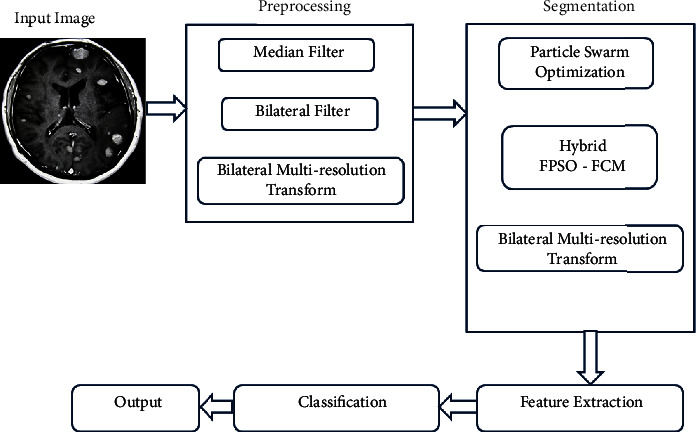
Proposed work.

The motivation of this study is discussed with (a) the uncertainty and impreciseness in detecting tumour formation, orientation, and location; (b) the use of the “Divide and conquer” strategy on MRI imaging to perform segmentation; (c) access to analysis and easily deployable tools such as MATLAB packages; (d) lacks powerful feature extraction through multiple orientations and efficient classification. The proposed work is shown in Figure 1.

## 2. Related Works

Biratu et al. has suggested utilising intersection over union, similarity score, and accuracy against the real world to evaluate ROI [6]. The ROIs were compared with several deep learning and region-based techniques. They generated a DSS value by randomly choosing an image to test. The seed point initiation and obtaining a better ROI for any photos of a brain tumour provide the key difficulties of this work of region developing segmentation method. The seed point was created to automatically produce for the major approaches of this study's random image selection. Shahvaran et al. has used multimodal tumour pictures to perform automatic [7] tumour extraction. Using the active contour approach, which is utilised for tumour disease extraction based on the border of the discovered brain tumour regions, it is possible to detect the tumour image using the *K*-means clustering algorithm. On the BraTS 2013 dataset, which included patients with both high-grade and low-grade tumours, the suggested technique was assessed. The suggested method outperformed existing active contour based methods when it came to segmenting tumours, with mean Dice similarity coefficients for high-grade and low-grade tumours of 0.9179 (0.025) and 0.8910 (0.042), respectively. Arun Kumar et al. [8] developed an outstanding performance in brain tumour classification and found the best accuracy rate. The classification techniques are built on the classic machine vision approaches, including image enhancement, Fourier transform, segmentation, HOG, ANN classification, and feature extraction. So, the classification method gives a 92.14% accuracy using *K*-fold cross validation techniques.

Breast cancer is a dreadful disease. Early detection of ductal carcinoma (breast cancer) will save lives. This is possible by amalgamating microwaves and infrared thermography using a Convolutional Neural Network (CNN). Microwaves and infrared thermography are used as [9, 10] sources of radiation and heat imaging recorders, respectively. By transmitting the radiant energy to the mammary gland with the electrical properties of normal and abnormal tissue, the magnetic permeability of normal tissue contradicts with the ill one, which has a radius of 5 mm, and its location can be found by placing a sensitive screen below the mamma. Hence, the heat distribution pattern can be traced out. This technique is enhanced by implementing a Machine Learning (ML) model. Therefore, even a slight variation in heat pattern can be detected.

Usually, BTs (brain tumours) are categorized using MRI (Magnetic Resonance Imaging). In this paper, two databanks are used in which Gaussian Convolutional Neural Network (GCNN) is applied [11]. The first dataset is used to group tumours into glioma, meningioma, and pituitary. With the second database, glioma is disparate into three grades. The first and second databases consist of 3064 and 516 images, correspondingly, that are elicited from *T*1-weighted complexity-improved images. The datasets are interconnected after performing GCNN. Using data augmentation, an accuracy of 97.14% in the first and 99.8% in the second databank is achieved.

## 3. Proposed Method

### 3.1. Preprocessing

#### 3.1.1. Adaptive Bilateral of Multi-Resolution Framework

A multiresolution analysis is the best tool for reducing noise in signals. So, it is more possible to differentiate image information, and noise is restored at one resolution level than another. As proposed, the adaptive bilateral method of the multiresolution framework is applied which decomposes a signal into frequency sub-bands with wavelet decomposition. A signal is back reconstructed; a bilateral method is applied to the sub-bands and the adaptive bilateral method of multiresolution was reducing low-frequency noise components. Applying wavelet threshold to the entire sub-bands, it is reducing the image noise very effectively in the combination of adaptive bilateral multi-resolution and wavelet threshold.

#### 3.1.2. Compression Blocking Artifacts Reduction

The two parameters of the spatial domain (*σ*_*d*_) and intensity domain (*σ*_*d*_) are the control and behavior of the standard bilateral technique. In this case, the parameters are chosen carefully for the compression artifact reduction.

### 3.2. Analysis of Parameter

The two parameters of the spatial domain (*σ*_*d*_) and intensity domain (*σ*_*d*_) are control and behaviour of the standard bilateral filter. In the case of a bilateral filter, parameters are chosen carefully for the compression artifact reduction.

The edge signal shows the first subplot; the edge discontinuity value is 10. The output of the intensity domain *σ*_*r*_ values shown in the second subplot in that figure. If the intensity domain *σ*_*r*_ value is less than the discontinuity, basically the filter is useless to reducing the discontinuity. If the intensity domain *σ*_*r*_ value is larger than the discontinuity, it reducing the discontinuity.

The *r* value in the spatial domain can be utilised to control and enhance smoothness. If the *d* value of the spatial domain is greater than the level of discontinuity, the smoothing will be more extensive. It is possible to lessen the discontinuity without using *d*'s solution if the spatial discontinuity is less than the value of *d*. As a result, we take measurements of the block boundaries, *r*'s value, and the degree of discontinuity. The texture region would be too smoothed if the *d* value were small; instead, it is huge. The nonadaptive bilateral filter will result in numerous issues, such as too blurred texture regions when a strong parameter is used to reduce the blockiest. The blocking artefacts are not there when we choose a lesser setting.

The control and behavior of the standard bilateral filter are controlled by the two parameters of the spatial domain (*d*) and the intensity domain (*d*). In the case of bilateral filter parameters are chosen carefully for compression artifact reduction.

The edge signal shows the first subplot; the edge discontinuity value is 10. The output of intensity domain *r* values is shown in the second subplot in that figure. If the intensity domain *r* value is less than the discontinuity, the filter is useless to reduce the discontinuity. If the intensity domain *r* value is larger than the discontinuity, it will reduce the discontinuity.

At that same time, enhanced smoothing can be controlled by the spatial domain *r* value. The spatial domain *d* value is larger than the discontinuity, the wider the extent of the smoothing. If the spatial domain *d* value is less than the discontinuity, then reducing the discontinuity has no solution. As a result, we measure the discontinuity amount along with the block boundaries and the value of *r* accordingly. To avoid over-smoothing the texture region, the _*d*_ value is large; otherwise, it is small. The nonadaptive bilateral filter will create some issues, like when we choose strong parameters to reduce the blockiest; the texture region is over-blurred. When we choose weaker parameters, it does not completely remove the blocking artifacts.

**Figure 2:**
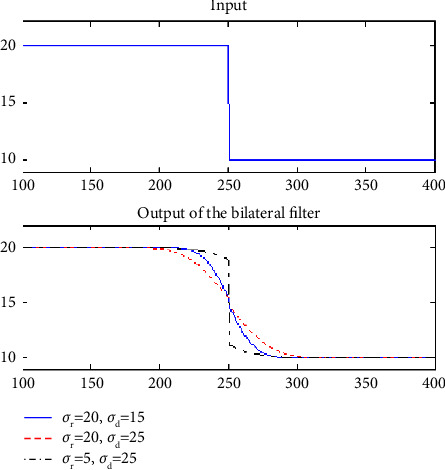
The parameter values of sigma_r and sigma_d on a block discontinuity.

**Figure 3:**
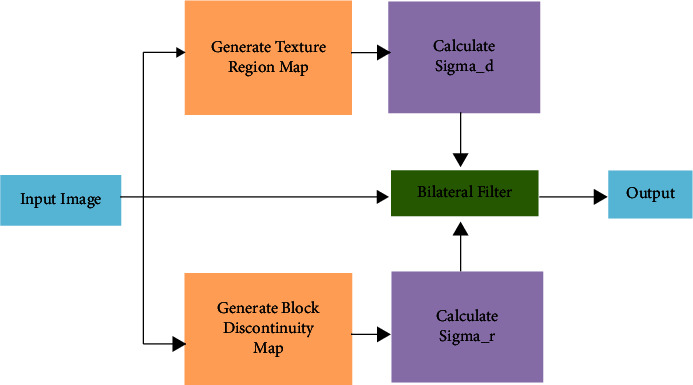
Framework of adaptive bilateral method.

Here, Figure 2 shows the parameter values of *σ*_*d*_ and *σ*_*d*_ on block discontinuity are illustrated. The input signal value is 10. The performance of the middle image shows the parameter *σ*_*d*_ value and different *σ*_*r*_ value. The performance of the last image shows *σ*_*r*_ value and different *σ*_*d*_ value. Previously we discussed, the two modules are included in this framework. The first module is detecting the discontinuity amount and adjusts the parameter *σ*_*r*_ value. The second module is detecting the smoothness of a texture regions and adjust the parameter *σ*_*d*_ value. The new framework of the adaptive bilateral method in Figure 3.

Here, Figure 2 shows the parameter values of *d* and *d* on block discontinuity are illustrated. The input signal value is 10. The performance of the middle image shows the parameter *d* value and different *r* value. The performance of the last image shows *r* value and different *d* value. Previously we discussed, the two modules are included in this framework. The first module is detecting the discontinuity amount and adjusts the parameter *r* value. The second module is detecting the smoothness of a texture regions and adjust the parameter *d* value. The new framework of the adaptive bilateral method in Figure 3.

To detect block, discontinuities, the vertical boundaries of an input image is filtered with the value of [−1, 0, 1] and the horizontal boundaries with [−1, 0, 1]^*T*^ the value of the results. The parameter *σ*_*r*_ should be at least equal to effective values. The block discontinuities are detected in the boundaries: if the bilateral filter is applied to the boundary only, the block cannot reduce. If we reduced the block very effectively, entire blocks of the bilateral filter should be applied.

### 3.3. Hybrid Segmentation

An enhanced EDPSO (Darwinian particle swarm optimization) and Quantum Entanglement and Wormhole Behaved Particle Swarm Optimization Techniques [12–14] were proposed to segment a tumour image which overcomes this existing method of GCPSO (Guaranteed Convergence Particle Swarm Optimization). We propose, a new Hybrid GCPSO (Guaranteed Convergence Particle Swarm Optimization)—FCM (Fuzzy C-Mean) algorithm use to each particle to every number of generations/iterations so the fitness value of all the particles to improve.

Hybrid GCPSO algorithm for fuzzy clustering is as follows:

1. Initializing the attributes of GCPSO and Fuzzy *c*-mean
2. Initializing the attributes of population *P*, *c*1, *c*2, *m*, and *w*
3. Generate particle attribute represent *P* (*x*, *g*best, *p*best, and velocity are *n∗c* matrices)

#### 3.3.1. GCPSO

- Compute the cluster center for the whole particles
- Compute the fitness value of all the particles
- Calculate *p*best for all the particles
- Evaluate *g*bestfor the swarm
- Entire particle velocity to be evaluated
- Entire particle position to be evaluated

#### 3.3.2. Fuzzy *C*-Mean

(i) Compute the center of the cluster for the each particles
(ii) Find the Euclidian Distance method to represent the attribute *d*_*ij*_, the *i* varies from 1, 2,…, *n*; and the *j* varies from 1, 2,…, *c*; for each particle using the following equation:
  (1) $$\begin{array}{c} d_{ij}=\left\|o_{i}-z_{j}\right\|\text{.} \end{array}$$
(iii) Evaluate of −++ the function *µ*_*ij*_, *i* varies from 1, 2,…, *n*; and the *j* varies from 1, 2,…, *c*; for the entire particle.
  (2) $$\begin{array}{c} \mu_{ij}=\frac{1}{\sum_{k=1}^{c}{\left(d_{ij}/d_{ik}\right)}^{2/m-1}}\text{.} \end{array}$$
(iv) All the particle of *p*best to be evaluated
(v) Swarm of *g*best to be evaluated
(vi) Goto step, If Fuzzy *C*-mean terminating state is not met
(vii) Goto step, If the GCPSO terminating state is not met

The GCPSO (Guaranteed Convergence Particle Swarm Optimization)-FCM (Fuzzy *C*-Mean) algorithm focuses on a new particle deal with a present best position in the neighbourhood. In the present research study, each element considers as a member of the swarm, the following a new particle for the velocity update equation is given as follows:

(3) $$\begin{array}{c} \nu \varphi \left(t+1\right)=x\varphi \left(t\right)+p\;best\left(t\right)+\omega v\varphi \left(t\right)+\rho \left(t\right)\left(1-2r\right)\text{.} \end{array}$$

To improve the random search area in the region of the best position was improved. The random search area of the random vector and random diameter *r* and *ρ*(*t*).

Here, the term ^#^sc is a success and ^#^fc is failure is defined as a number of consecutive failure and success. Since high dimensional search spaces in a few iteration sc values are 15 and fc value is 5.

### 3.4. Enhanced Fuzzy *C*-Mean Techniques

FCM is clustering methods which allows for partitioning into multiple clusters. The minimization of the objective function:

(4) $$\begin{array}{c} J_{m}=\overset{N}{\underset{i=1}{\sum }}\overset{C}{\underset{j=1}{\sum }}u_{ij}^{m}{\left\|x_{i}-c_{j}\right\|}^{2}\text{,}1\leq m<\infty , \end{array}$$

where *m* = actual number higher than the value of 1. *u*_*ij*_ = *x*_*i*_ is a degree of partisanship in cluster *j*, Now the *x*_*i*_ is the number of *i*^th^*d*-dimensional measured data. *c*_*j*_ = Cluster center.

Enhanced Fuzzy *C*-Mean technique is used to calculate the middle of the cluster for entire particles and the Euclidian Distance method *d*_*ij*_, the *i* values are 1, 2,…, *n*; and the *j* values are 1, 2,…, *c*; the following equation is

(5) $$\begin{array}{c} d_{ij}=\left\|o_{i}-z_{j}\right\|\text{.} \end{array}$$

To upgrade the function *µ*_*ij*_, *i* values are 1, 2,…, *n*; and the *j* values are 1, 2,…, *c*; for the entire particle. The following equation is

(6) $$\begin{array}{c} \mu_{ij}=\frac{1}{\sum_{k=1}^{c}{\left(d_{ij}/d_{ik}\right)}^{2/m-1}}\text{.} \end{array}$$

Finally, evaluate the best position (*p*best) for entire particles and evaluate the global best position (*g*best) for the swarm. If Fuzzy C-Mean Clustering is the termination state GOTO initial stage of the Fuzzy C-Mean Clustering and If the GCPSO terminating state is not met, GOTO initial stage of the method. In the current research, 130 different brain tumour test photos are analyzed. Utilizing the suggested approach of multiresolution of the adaptive bilateral technique, reduce noise and improve image quality. In this research work, the proposed hybrid segmentation approach of Guaranteed Convergence Particle Swarm Optimization (BMPSO) and Fuzzy *C*-Mean (FCM) was compared and found to be the more effective method. This new framework, a hybrid BMPSO-FCM, has assessed an accuracy rate of 95.92 percent for detecting brain tumour cells.

Finally, evaluate the optimal particle position (*p*best) and the optimal global particle position (*g*best) for the swarm. If Fuzzy *C*-Mean Clustering is in the termination state, proceed to the initial stage of Fuzzy *C*-Mean Clustering. Similarly, if Guaranteed Convergence Particle Swarm Optimization is in the termination state, proceed to the initial stage of the procedure. In the current study, 130 distinct test images of brain tumours are analyzed. Reduce noise and enhance image quality by implementing the suggested multi-resolution adaptive bilateral technique. In this study, the hybrid segmentation methodology of Guaranteed Convergence Particle Swarm Optimization (BMPSO) and Fuzzy *C*-Mean (FCM) was determined to be the superior method. This new framework, a hybrid BMPSO-FCM, offers a 95.92 percent detection accuracy rate for brain tumour cells.

### 3.5. Performance Measures

The image segmentation metrics are divided into region-based and boundary-based methods. A different performance metrics are used to evaluate in the segmented image as follows:

- True Positive Rate (TPR): an outcome of this model to predict the positive class correctly
- True Negative Rate (TNR): an outcome of this model to predict the negative class correctly
- False Positive Rate (FPR): an outcome of this model to predict the positive class incorrectly
- False Negative Rate (FPR): an outcome of this model to predict the negative class incorrectly.

## 4. Experimental Result

**Figure 4:**
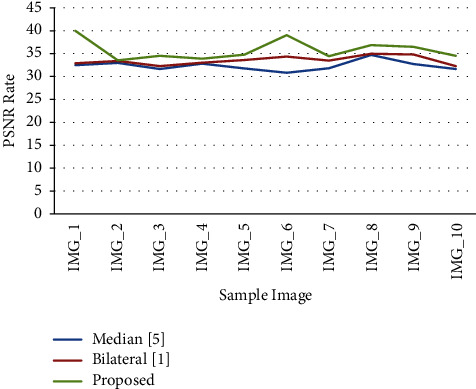
A PSNR rate performance comparison of various methods.

A multiresolution analysis is the most important tool for reducing noise in signals. It is possible to differentiate image information and noise is better than one resolution level than another. As proposed, the adaptive bilateral method of the multiresolution framework is applied it is shown in Figure4, decomposed a signal into frequency sub-bands with wavelet decomposition. Based on these trials, the findings were reviewed with prior techniques utilising the bilateral method's parameters of *d* = 1.8, *r* = 2*∗n*, and image size 7 × 7. The adaptive bilateral filter technique was compared to earlier techniques. In this proposal, the parameter values should be set to *d* = 1.8, *r* = 1.0*∗n*, and the image size should be 11 × 11 at each level.

The proposed adaptive bilateral approach was found to produce better results when the MSE rate and PSNR values were compared with those of earlier methods.

To modify the various analyses of the original picture resolution in order to remove the noise from the original image. In addition, it is possible to separate picture information from noise information, which is preferred from one resolution level to another. For the various image resolutions, wavelet thresholding and the adaptive bilateral technique were combined. To minimise low-frequency noise components, the adaptive bilateral technique was used to sub-bands. Refer to Table 1, which is shown in the input image with noise and discussed with the preceding section, to choose the parameter values for the standard bilateral filter, *d* = 1.8 and *r* = 2.

**Figure 5:**
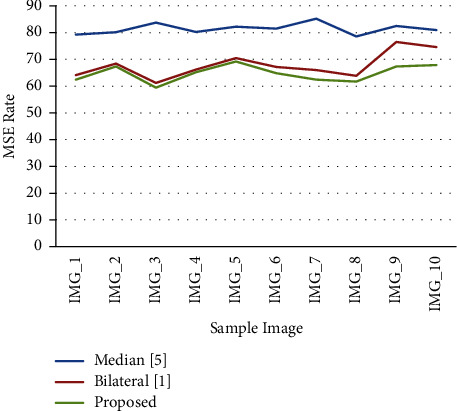
A MSE rate performance comparison of various methods.

Table 1 demonstrates a comparison of several filtering techniques in order to identify the improved accuracy of the suggested approach. The adaptive bilateral filter is shown in Figure 5 to have a better MSE (Mean Square Error) rate when preprocessing approaches.

The new method's superior accuracy rate in comparison to the median filter and bilateral filter was demonstrated by a graphical view examination of earlier techniques.

A comparison of several filtering techniques is shown in Table 2 in order to identify the outcomes of the suggested method that have higher accuracy. The PSNR rate of the adaptive bilateral filter was demonstrated by comparing the filter method.

The new method's superior accuracy rate in comparison to the median filter and bilateral filter was demonstrated by a graphical view examination of earlier techniques shown in Figure 4.

### 4.1. Experiment Result for Hybrid GCPSO—FCM

Performance of the proposed image segmentation method of hybrid GCPSO-FCM where the more accuracy rate to find the tumour identification.

The image segmentation accuracy rate was measured using ^#^TPR, ^#^TNR, ^#^FPR, and ^#^FNR by comparing various algorithms with manual results.

In Table 3 demonstrates a comparison of several filtering techniques in order to identify the improved accuracy of the suggested approach. The image segmentation metrics are divided into region-based and boundary-based methods. A different performance metrics are used to evaluate in the segmented image as TP, TN, FP, and FN.

In Table 4, we found the performance measures of the proposed image segmentation method of hybrid GCPSO-FCM where the more accuracy rate to find the tumour detection in Statistical analysis of FPSO-FCM Algorithm.

Table 5 demonstrates a comparison of several filtering techniques in order to identify the improved accuracy of the suggested approach. The image segmentation metrics are divided into region-based and boundary-based methods. A different performance metrics are used to evaluate in the segmented image as TP, TN, FP, and FN. We propose, a new Hybrid GCPSO-FCM algorithm use to each particle to every number of generations/iterations so the fitness value of all the particles to improve the Accuracy rate.

Table 6 shows that the performance comparison of various segmentation algorithm PSO and FPSO-FCM accuracy rate to be compared with the proposed technique GCPSO-FCM was better accuracy rate to detect the early detection.

**Figure 6:**
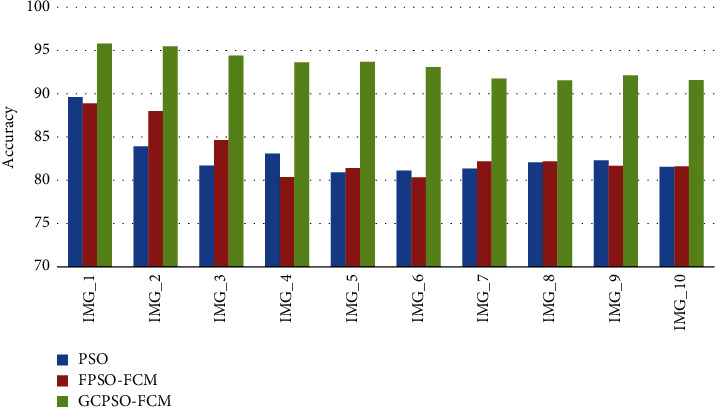
Comparative analysis of accuracy value.

Figure 6 shows the graphical view analysis of various segmentation algorithm PSO and FPSO-FCM accuracy rate to be compared with the proposed technique GCPSO-FCM was better accuracy rate to detect the early detection.

In Feature Extraction, using the four GLCM features that are measured above are computed using applied techniques and are examined. The structure exhibits substantial image intensity changes using contrast measurement. The four segmentation strategies that have been suggested offer a maximum accuracy rate of 95% compared to the limitations of the current algorithm. This study uses GLCM features and a matrix to analyze statistical texture in images of brain tumours. In order to determine four directions such as 0°, 45°, 90°, and 135°, the Feature Analysis measures four texture parameters, including energy, contrast, correlation, and homogeneity with two picture intensities, 128 and 256. One of the four GLCM features, strip str2, has a high intensity of 569 for angle 45° in the average contrast, whereas angle 0° has the lowest value of the contrast.

The correlation measures the correlation between neighbouring pixels, which spans from −1 to 1. In addition to the value of energy, the count of homogeneity aids the correlation because it is high in the 0° angle. When the image intensity is reduced, the correlation and homogeneity values do not decrease as much as the contrast value.

In addition, the energy has a high value when measured vertically. In comparison to other angles, the angle of 0° has a higher energy value. When the contrast value was decreased from 256 to 128, so was the image intensity. Even though the picture intensity count is decreased, the values of energy, correlation, and homogeneity have not altered shown in Table 7.

In Classification, the five types of kernel functions using SVM classification. The polynomial kernel function is tested from 1 to 5°, and the scaling factor is tested from 1 to 5 in the kernel function of Gaussian Radial Based Function, ANOVA kernel, and Exponential Radial Based Function. The performance of the present research method was evaluated in form of sensitivity, specificity, and accuracy.

The performance measures the SVM classification with GRBF kernel function is giving a better accuracy rate in this present research work and it also gives 98.83% of sensitivity, 90% of specificity, and a value of accuracy of 95.32% shown in Table 8.

Table 9 shows the performance comparison of various hybrid techniques to detect the early detection of brain tumours, the proposed technique GCPSO-FCM was compared and proved with a better accuracy rate to detect the early detection.

In that Table 10 shows the result analysis of various hybrid techniques to compare proved with the proposed techniques. The proposed techniques was shows the improved accuracy rate.

## 5. Conclusion

With the findings of this study, we offer an effective technique for improving image quality, eliminating noise in the actual image, and locating, and limiting boundary and texture discontinuities to adjust or take control of the spatial and intensity parameters in a bilateral method that results in a higher MSE and PSNR value. Low-frequency sub-bands can benefit from the wavelet threshold and adaptive bilateral filter combination. We suggested a hybrid segmentation technique that combines GCPSO—FCM (Fuzzy *C*-Mean) and achieves 95.32% accuracy. The four segmentation strategies that have been suggested offer a maximum accuracy rate of 95% compared to the limitations of the current algorithm. This study uses GLCM features and a matrix to analyze statistical texture in images of brain tumours. In order to determine four directions such as 0°, 45°, 90°, and 135°, the Feature Analysis measures four texture parameters, including energy, contrast, correlation, and homogeneity with two picture intensities, 128 and 256. One of the four GLCM features has a high intensity of 569 for angle 45° in the average contrast, whereas angle 0° has the lowest value of the contrast.

The correlation measures the correlation between neighbouring pixels, which span from −1 to 1. In addition to the value of energy, the count of homogeneity aids the correlation because it is high in the 0° angle. When the image intensity is reduced, the correlation and homogeneity values do not decrease as much as the contrast value. Finally, it has been demonstrated that the GCPSO—FCM utilising an SVM classification methodology is suitable for categorising the image as malignant and noncancerous through the comparison of graphical view analysis of prior methods. In addition to having a 95.92% accuracy rate, SVM classification with the GRBF kernel function also has 99.53% sensitivity and 90% specificity.

## Figures and Tables

**Table 1:** A MSE rate performance comparison of pre-processing.

Input	Median	Bilateral	Proposed
IMG_1	79.214	64.03	61.327
IMG_2	80.131	68.371	67.349
IMG_3	83.722	67.191	64.457
IMG_4	80.219	66.147	65.124
IMG_5	82.191	70.492	69.191
IMG_6	81.41	67.12	64.81
IMG_7	85.13	65.99	63.47
IMG_8	78.51	66.14	64.14
IMG_9	82.42	76.41	67.18
IMG_10	80.94	74.51	67.85

**Table 2:** A PSNR rate performance comparison of preprocessing.

Sample-image	[5] Median	[1] Bilateral	Proposed
IMG_1	32.46	32.85	39.92
IMG_2	32.94	33.43	33.56
IMG_3	31.61	32.22	34.52
IMG_4	32.81	33.01	33.89
IMG_5	31.77	33.61	34.77
IMG_6	30.82	34.35	39.01
IMG_7	31.81	33.49	34.44
IMG_8	34.71	34.97	36.84
IMG_9	32.73	34.81	36.47
IMG_10	31.63	32.27	34.51

**Table 3:** Statistical analysis of PSO algorithm.

Sample image	TP	TN	FP	FN	Accuracy rate
IMG_1	86.541	89.120	8.880	11.459	89.623
IMG_2	83.634	88.400	13.601	19.366	83.919
IMG_3	82.257	81.145	18.855	17.743	81.701
IMG_4	84.632	81.507	18.493	15.368	83.070
IMG_5	80.934	80.909	19.091	19.066	80.921
IMG_6	80.812	81.422	18.578	19.188	81.117
IMG_7	80.858	81.814	18.186	19.142	81.336
IMG_8	79.139	85.008	14.992	20.861	82.074
IMG_9	79.466	85.157	14.843	20.535	82.311
IMG_10	77.783	85.345	14.655	22.217	81.564

**Table 4:** Statistical analysis of FPSO-FCM algorithm.

Sample image	TP	TN	FP	FN	Accuracy rate
IMG_1	87.465	90.327	9.673	12.535	88.896
IMG_2	86.195	89.813	10.187	13.805	88.004
IMG_3	82.902	86.394	13.606	17.098	84.648
IMG_4	80.327	80.385	19.615	19.673	80.356
IMG_5	82.419	80.392	19.608	17.582	81.405
IMG_6	81.077	79.597	20.404	18.923	80.337
IMG_7	79.466	84.822	15.178	20.394	82.202
IMG_8	79.466	84.822	15.178	20.394	82.202
IMG_9	81.879	81.590	18.410	18.269	81.674
IMG_10	77.768	85.428	14.572	22.232	81.598

**Table 5:** Statistical analysis of GCPSO-FCM algorithm.

Sample image	TP	TN	FP	FN	Accuracy rate
IMG_1	91.616	99.999	0.001	8.384	95.807
IMG_2	90.956	99.999	0.001	9.044	95.478
IMG_3	88.840	99.999	0.001	11.159	94.420
IMG_4	87.295	99.999	0.001	12.705	93.647
IMG_5	87.358	99.999	0.001	12.642	93.679
IMG_6	86.1567	99.999	0.001	13.843	93.078
IMG_7	83.487	99.999	0.001	16.513	91.743
IMG_8	83.108	99.999	0.001	16.892	91.554
IMG_9	84.291	99.999	0.001	15.709	92.145
IMG_10	83.192	99.999	0.001	16.808	91.595

**Table 6:** Comparison of accuracy value using various segmentation algorithms.

Sample image	PSO	FPSO-FCM	GCPSO-FCM
IMG_1	89.623	88.896	95.807
IMG_2	83.919	88.004	95.478
IMG_3	81.701	84.648	94.42
IMG_4	83.07	80.356	93.647
IMG_5	80.921	81.405	93.679
IMG_6	81.117	80.337	93.078
IMG_7	81.336	82.202	91.743
IMG_8	82.074	82.202	91.554
IMG_9	82.311	81.674	92.145
IMG_10	81.564	81.598	91.595

**Table 7:** GLCM texture feature.

Angular of image intensity	Offset		Contrast	Correlation	Energy	Homogeneity
128, 0°	0	1	61.2	0.96	0.019	0.9
128, 45°	−1	1	138	0.9	0.017	0.62
128, 90°	−1	0	95.1	0.94	0.015	0.73
128, 135°	−1	−1	94.3	0.94	0.014	0.68
256, 0°	0	1	289	0.96	0.019	0.78
256, 45°	−1	1	569	0.9	0.017	0.54
256, 90°	−1	0	379	0.94	0.015	0.68
256, 135°	−1	−1	374	0.94	0.014	0.63

**Table 8:** Performance measures various kernel functions using the SVM classifier.

Kernel	Sensitivity	Specificity	Accuracy
Linear	94.00	73.27	92.34
Polynomial	96.02	85.00	93.51
GRBF	**98.83**	**90.00**	**95.32**
ERBF	97.05	77.77	91.93
ANOVA	98.69	84.21	92.09

**Table 9:** Comparison of accuracy value of classification techniques.

Techniques	(%) Accuracy
[4] PSO, GA and SVM	87.3
[6] FPSO-FCM and SVM	91.25
Proposed GCPSO-FCM and SVM	**95.32**

**Table 10:** Ground truth analysis of brain MRI.

Pre-processing	Segmented image	Truth image	Comparison	Accuracy result
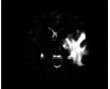	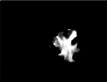	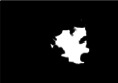	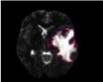	85.51
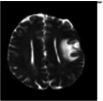	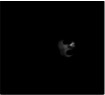	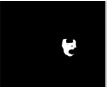	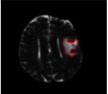	87.3

PSO, GA and SVM				
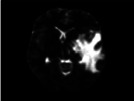	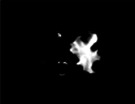	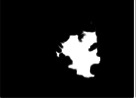	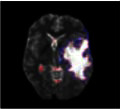	91.25
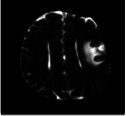	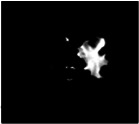	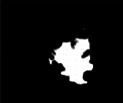	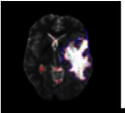	90.53

GCPSO-FCM and SVM				
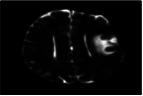	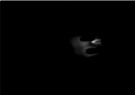	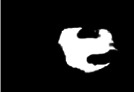	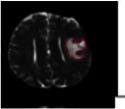	93.62
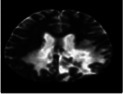	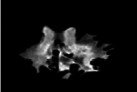	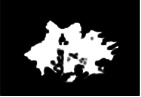	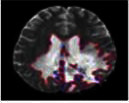	95.32

## Data Availability

The data used to support the findings of this study are included within the article.

## References

[1] Militello C., Rundo L., Dimarco M. (2022). Semi-automated and interactive segmentation of contrast-enhancing masses on breast DCE-MRI using spatial fuzzy clustering. *Biomedical Signal Processing and Control*.

[2] Ilhan U., Ilhan A. (2017). Brain tumor segmentation based on a new threshold approach. *Procedia Computer Science*.

[3] Tamal M. (2020). Intensity threshold based solid tumour segmentation method for Positron Emission Tomography (PET) images: a review. *Heliyon*.

[4] Rundo L., Militello C., Vitabile S. (2016). Combining split-and-merge and multi-seed region growing algorithms for uterine fibroid segmentation in MRgFUS treatments. *Medical, & Biological Engineering & Computing*.

[5] Amin J., Anjum M. A., Gul N., Sharif M. (2022). A secure two-qubit quantum model for segmentation and classification of brain tumor using MRI images based on blockchain. *Neural Computing & Applications*.

[6] Biratu E. S., Schwenker F., Debelee T. G., Kebede S. R., Negera W. G., Molla H. T. (2021). Enhanced region growing for brain tumor MR image segmentation. *Journal of Imaging*.

[7] Shahvaran Z., Kazemi K., Fouladivanda M., Helfroush M. S., Godefroy O., Aarabi A. (2021). Morphological active contour model for automatic brain tumor extraction from multimodal magnetic resonance images. *Journal of Neuroscience Methods*.

[8] Arun Kumar N., Mohammed M. A., Mostafa S. A., Ibrahim D. A., Rodrigues J. J., Albuquerque V. H. C. (2020). Fully automatic model‐based segmentation and classification approach for MRI brain tumor using artificial neural networks. *Concurrency and Computation: Practice and Experience*.

[9] Sangeetha S. K. B., Kumar M. S., Rajadurai H., Rajadurai H., Maheshwari V., Dalu G. T. (2022). An empirical analysis of an optimized pretrained deep learning model for COVID-19 diagnosis. *Computational and Mathematical Methods in Medicine*.

[10] Kogilavani S. V., Prabhu J., Sandhiya R. (2022). COVID-19 detection based on lung CT scan using deep learning techniques. *Computational and Mathematical Methods in Medicine*.

[11] Batchelor B. G. (2012). *Machine Vision Handbook*.

[12] Vijay V., Kavitha A. R., Rebecca S. R. (2016). Automated brain tumor segmentation and detection in MRI using enhanced Darwinian particle swarm optimization (EDPSO). *Procedia Computer Science*.

[13] Zhang T., Zhang J., Xue T., Rashid M. H. (2022). A brain tumor image segmentation method based on quantum entanglement and Wormhole behaved particle swarm optimization. *Frontiers of Medicine*.

[14] Nawaz S. A., Khan D. M., Qadri S. (2022). Brain tumor classification based on hybrid optimized multi-features analysis using magnetic resonance imaging dataset. *Applied Artificial Intelligence*.

